# Oxidative Stress Markers in Obsessive-Compulsive Disorder

**DOI:** 10.1192/j.eurpsy.2023.1957

**Published:** 2023-07-19

**Authors:** A. Maia, J. Oliveira, N. Descalço, B. Barahona-Corrêa, A. J. Oliveira-Maia

**Affiliations:** 1 Neuropsychiatry Unit, Champalimaud Foundation; 2 Nova Medical School, NOVA University Lisbon; 3 Department of Psychiatry and Mental Health, Centro Hospitalar de Lisboa Ocidental, Lisbon; 4 Psychiatry and Mental Health Department, Hospital Garcia de Orta, Almada, Portugal

## Abstract

**Introduction:**

Obsessive–compulsive disorder (OCD) is a chronic, prevalent, and highly impairing psychiatric illness. Although the pathophysiology of OCD remains unknown, pathways involved in oxidative stress (OS) have been implicated. However, the complete clinical picture has been rarely considered, and it remains unclear whether oxidative dysregulation is inherent to OCD pathophysiology, or whether it is a consequence of confounding factors such as age, body mass index (BMI) or smoking.

**Objectives:**

In this work, we aim to assess oxidant and antioxidant markers and its clinical correlates in a well characterized sample of patients with OCD and controls, to test the hypothesis that altered OS markers are associated with OCD, rather than to illness-related behavioral changes or comorbidities.

**Methods:**

60 patients with OCD and 60 age and sex-matched control volunteers were recruited and assessed for sociodemographic and clinical variables using the Yale-Brown Obsessive-Compulsive Scale-II, the Beck Depression Inventory-II and the State-Trait Anxiety Inventory and Mini International Neuropsychiatric Interview. Three oxidant [8-hydroxy-2’-deoxyguanosine (8-OhdG), malondialdehyde, protein carbonyl] and three antioxidant [catalase, glutathione-peroxidase and superoxide dismutase (SOD)] markers were assessed in serum using Enzyme-Linked Immunosorbent Assay (ELISA). After comparing between groups, the association between OS markers and OCD characteristics, psychiatric medication and psychiatric comorbidities was assessed among patients with OCD. All analyses were adjusted for BMI, smoking and presence of physical comorbidities.

**Results:**

The six OS markers were similar between patients with OCD and controls. Among patients with OCD, patients with more obsessive and depressive symptoms had lower concentrations of 8-OHdG, although this correlation may be sensitive to extreme values. Also, those who were on higher doses of antidepressants had lower concentrations of SOD. The remaining OS markers were not associated with OCD characteristics, psychiatric medication, or comorbidities.

**Conclusions:**

Our results suggest that OS markers in blood do not seem to be a good biomarker of disease in symptomatic adult patients with OCD, and that OCD characteristics and comorbidities do not seem to have a clear impact on OS profile. Several factors contribute to the robustness of our findings, namely the sample size, the adjustment for confounding factors, and the assessment of a representative panel of OS markers using strict experimental methods. Future studies should always control for confounding factors when assessing OS markers and study OS profile in more specific samples, such as children or treatment-naïve patients.

**Disclosure of Interest:**

None Declared

